# TMAO Upregulates Members of the miR-17/92 Cluster and Impacts Targets Associated with Atherosclerosis

**DOI:** 10.3390/ijms232012107

**Published:** 2022-10-11

**Authors:** Laura Díez-Ricote, Paloma Ruiz-Valderrey, Víctor Micó, Ruth Blanco, Joao Tomé-Carneiro, Alberto Dávalos, José M. Ordovás, Lidia Daimiel

**Affiliations:** 1 Nutritional Control of the Epigenome Group, Precision Nutrition and Obesity Program, IMDEA Food, UAM + CSIC, 28049 Madrid, Spain; 2 Research and Development Department, Biosearch Life Company, 28031 Madrid, Spain; 3 Epigenetics of Lipid Metabolism Group, Precision Nutrition and Cardiometabolic Health Program, IMDEA Food, UAM + CSIC, 28049 Madrid, Spain; 4 Nutrition and Genomics Laboratory, JM_USDA Human Nutrition Research Center on Aging, Tufts University, Boston, MA 02111, USA

**Keywords:** cardiovascular disease, TMAO, microRNAs, atherosclerosis, inflammation

## Abstract

Atherosclerosis is a hallmark of cardiovascular disease, and lifestyle strongly impacts its onset and progression. Nutrients have been shown to regulate the miR-17/92 cluster, with a role in endothelial function and atherosclerosis. Choline, betaine, and L-carnitine, found in animal foods, are metabolized into trimethylamine (TMA) by the gut microbiota. TMA is then oxidized to TMAO, which has been associated with atherosclerosis. Our aim was to investigate whether TMAO modulates the expression of the miR-17/92 cluster, along with the impact of this modulation on the expression of target genes related to atherosclerosis and inflammation. We treated HepG-2 cells, THP-1 cells, murine liver organoids, and human peripheral mononuclear cells with 6 µM of TMAO at different timepoints. TMAO increased the expression of all analyzed members of the cluster, except for miR-20a-5p in murine liver organoids and primary human macrophages. Genes and protein levels of SERPINE1 and IL-12A increased. Both have been associated with atherosclerosis and cardiovascular disease (CDVD) and are indirectly modulated by the miR-17-92 cluster. We concluded that TMAO modulates the expression of the miR-17/92 cluster and that such modulation could promote inflammation through IL-12A and blood clotting through SERPINE1 expression, which could ultimately promote atherosclerosis and CVD.

## 1. Introduction

Nutrition plays a crucial role in the development of non-communicable diseases and overall health. Approximately 45% of deaths caused by cardiometabolic events are associated with poor lifestyle habits and nutrition [1]. Saturated fats and red meat have previously been associated with cardiovascular disease (CVD); however, their specific involvement in its progression has not yet been established [2]. Indeed, some meta-analyses have not found any relationship between intake of red and processed meat and CVDs and all-cause mortality [3].

Choline, betaine (a metabolite from choline), and L-carnitine are abundant in eggs, fish, and meat. They can be metabolized into trimethylamine (TMA) by the gut microbiome. TMA is then absorbed and transported to the liver, where it is metabolized to trimethylamine-n-oxide (TMAO) by the flavin-containing mono-oxygenase (FMO) enzyme [4]. This metabolite has gained attention regarding its link to CVD [5] and has been reported to be a potential CVD marker linked to atherosclerosis and atheroma plaque formation [6]. Plasma TMAO levels could aid in predicting cardiovascular events, even when other biomarkers are within normal ranges [5]. Furthermore, mice supplemented with L-carnitine showed higher TMAO plasma levels and increased atherosclerosis [4], and APOE−/− mice fed with a high-fat diet and TMAO had significantly increased atherosclerotic plaque progression [7]. In addition, TMAO plasma levels are higher in subjects with myocardial infarction and are associated with a higher coronary atherosclerotic load [8]. However, high plasma TMAO levels have also been found in subjects with a healthy diet [9]. In addition, TMAO has not been associated with cardiovascular disease in children and their parents, although its precursors (i.e., choline, carnitine, and betaine) have been [10]. A similar finding was reported in the population from the CARDIA study, where TMAO levels were not associated with measures of atherosclerosis risk in young males and females [11]. Furthermore, TMAO and its relationships with CVDs have been studied in populations at high risk of suffering CVDs, and the debate is still open, so it can be suggested that concentrations of TMAO precursors could be more useful as a biomarker of CVD for the general population. Indeed, TMA has been proposed as a more precise and accurate marker of inflammation and CVD risk, as it has been reported that TMA levels are increased in cardiovascular patients compared to healthy subjects, while no such differences were observed in TMAO [12]. Thus, the TMA/TMAO ratio may be useful when evaluating these metabolites as CVD markers, as might the functionality of the FMO3 enzyme, which is responsible for the oxidation of TMA to TMAO. Very recently, we demonstrated that an increase in the intake of choline or betaine—precursors of TMAO—improved cardiometabolic and renal traits in a population of adult men and women with overweight/obesity and metabolic syndrome in the PREDIMED-Plus study [13]. Another previous study conducted by our team showed that TMAO increases the expression of miR-30c, with a role in lipid metabolism [14,15], and miR-21, which is fundamental in the resolution of acute inflammation [15,16]. In this study, we demonstrated the miR-30c-mediated regulation of PER2—a key circadian regulator—by TMAO and suggested a pro-inflammatory effect of TMAO. Therefore, results are contradictory and, consequently, the effects of TMAO on atherogenesis and cardiometabolic traits need to be further defined.

In recent years, more studies have highlighted the role of the miR-17/92 cluster in cardiovascular physiology [17]. Its dysregulation has been associated with arrhythmogenesis and cardiomyopathy [18], aberrant cardiomyocyte differentiation [19], myocardial infarction [20], and cardiac aging [21]. In addition, this cluster is also involved in lipid metabolism [22,23]. Moreover, our previous studies show that members of this cluster are modulated by diet—specifically, by the consumption of extra-virgin olive oil [24] and beer [25].

Given our previous results and the existing literature, we aimed to evaluate whether TMAO could modulate the expression of three microRNAs from the miR-17-92 cluster (i.e., miR-17-5p, miR-20a-5p, and miR-92a-3p) and of relevant targets involved in atherosclerosis progression and CVD, in two cellular models that are important in the biology of inflammation and lipid metabolism (i.e., macrophages and hepatocytes).

## 2. Results

**Figure 1:**
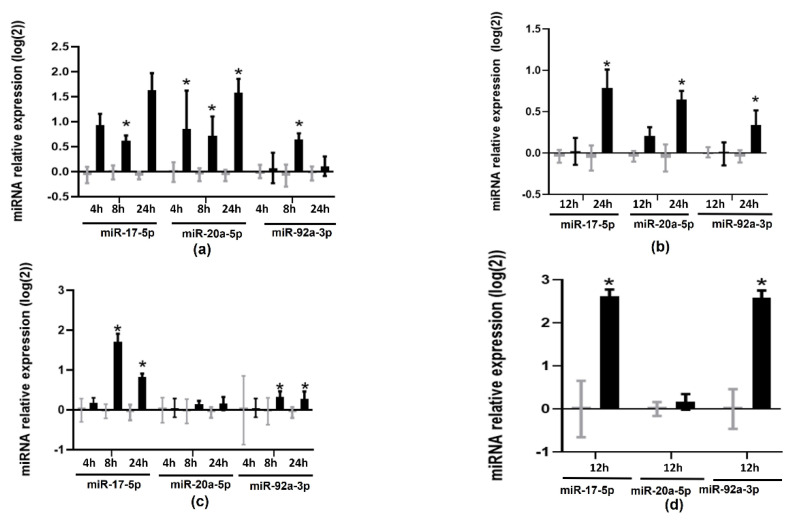
Effects of TMAO on the expression of miR-17-5p, miR-20a-5p, and miR-92a: (**a**) HEPG-2 cells, (**b**) THP-1 cells, (**c**) murine liver organoids, and (**d**) human primary macrophages were treated with 6 µM of TMAO at specified timepoints; miR-17-5p, miR-20a-5p, and miR-92a-3p levels were measured by RT-qPCR. mRNA levels were calculated with the 2^−ΔΔCT^ method and Log_2_-transformed. The mean ± SEM of three independent experiments is shown. Expression values in TMAO-incubated samples (black bars) were compared with corresponding control samples (grey bars) at each timepoint using Student’s *t*-test; * *p* < 0.05.

To test whether TMAO modified the expression of members of the miR17-92 cluster, we treated cells with 6 µM of TMAO at different times of incubation and analyzed the expression of miR-17-5p, miR-20a-5p, and miR-92a-3p by RT-qPCR. TMAO upregulated miR-17-5p (0.61 ± 0.10 at 8 h and 1.63 ± 0.34 at 24 h in HEPG-2 cells, and 0.79 ± 0.21 at 24 h in THP-1 cells) and miR-92a-3p (0.64 ± 0.12 at 8 h in HEPG-2 cells and 0.34 ± 0.17 at 24 h in THP-1 cells) (Figure 1a,b). These microRNAs were also upregulated in primary human macrophages (miR-17-5p 2.62 ± 0.15; miR-92a-3p 2.59 ± 0.16) and murine liver organoids (miR-17-5p 1.71 ± 0.19 at 8 h and 0.82 ± 0.08 at 24 h; miR-92a-3p 0.32 ± 0.14 at 8 h and 0.27 ± 0.19 at 24 h) (Figure 1c,d). However, miR-20a-5p was only upregulated in HEPG-2 (0.85 ± 0.76, 0.72 ± 0.38, and 1.58 ± 0.27 at 4, 8, and 24 h, respectively) and THP-1 cells (0.65 ± 0.10 at 24 h), but not in human primary macrophages or murine liver organoids (Figure 1c,d).

**Figure 2:**
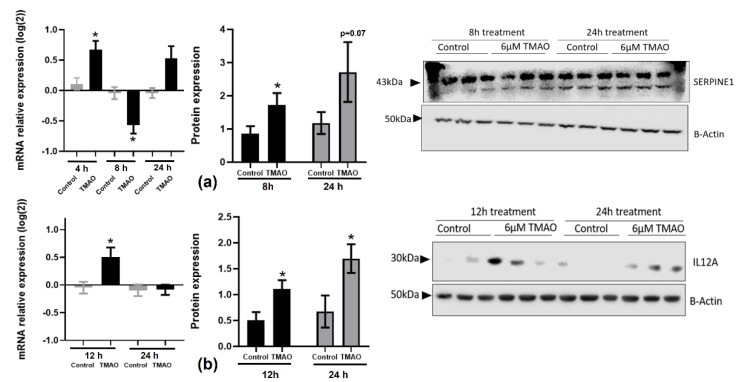
Effects of TMAO on SERPINE1 and IL-12A gene and protein levels: (**a**) Cells were treated with 6 µM of TMAO for 4 h, 8 h, and 24 h to measure *SERPINE1* gene expression and for 8 h and 24 h to measure SERPINE1 protein levels in HEPG-2 cells. (**b**) Levels of IL-12A were measured in THP-1 cells at 12 h and 24 h. Gene expression was measured by RT-qPCR and calculated with the 2^−ΔΔCT^ method and Log_2_-transformed. Protein levels were measured by Western blotting. Band density was measured and normalized with β-actin. The mean ± SEM of three independent experiments is shown. Expression values in TMAO-incubated samples (black bars) were compared with corresponding control samples (grey bars) at each timepoint using Student’s *t*-test; * *p* < 0.05.

We then searched the literature for targets of these microRNAs that were involved in regulating inflammation, endothelial or cardiac function, and the formation and progression of atheroma plaques. Among the putative targets, we selected SERPINE1 and IL-12 for further study due to their key role in the progression of atherosclerosis and the resolution of inflammation, respectively [26,27,28]. The miR-17-92 cluster indirectly targets *SERPINE1* and inhibits its expression by modifying the *PDLIM5* gene regulation involved in translocation of kinase to the muscle and cardiomyocyte contraction [29]. In addition, SERPINE1 could inhibit smooth muscle cell proliferation from arteries, stimulated by the miR-17/92 cluster. In neuroblastoma cells, it has been observed that when expression of the miR-17/92 cluster was activated, both *TGF-β* and *SERPINE1* were inhibited [30]. We found that *SERPINE1* gene expression was significantly increased and then decreased after 4 h (0.67 ± 0.14) and 8 h (−0.57 ± 0.14) of treatment, respectively, in HEPG-2 cells (Figure 2a). On the other hand, SERPINE1 protein levels were increased at both 8 h (1.73 ± 0.35) and 24 h (2.71 ± 0.9).

We observed a significant increase in IL-12A gene expression levels in THP-1 cells incubated with 6 µM of TMAO for 12 h (0.50 ± 0.17), whereas protein levels were significantly increased at the 12 h (1.11 ± 0.16) and 24 h (1.70 ± 0.27) timepoints (Figure 2b).

## 3. Discussion

This study shows how TMAO, at a physiological dose (6 µM), modulates the expression of the miR-17/92 cluster in two cell lines (HEPG-2 and THP-1). Additionally, we validated our results using two non-immortalized cellular models whose characteristics are likely to closely resemble what occurs in vivo, i.e., murine liver organoids and human primary macrophages. We found that miR-17-5p and miR-92a-3p were upregulated after TMAO exposure in HEPG-2 cells and liver organoids at 8 h and 24 h, and in THP-1 cells and human macrophages at 24 h. However, miR-20a-5p did not change in the murine liver organoids or human primary macrophages.

The miR-17-92 cluster, also known as oncomiR1 because of its role in the development of cancer, modulates energy metabolism in cancer cells. It has been shown that the lack of miR-92a upregulates glycolytic and oxidative metabolism in cancer cells; however, the lack of miR-17 and miR-20a inhibits glycolytic and oxidative metabolism as well as mTOR pathways in cancer cells and increases the AMPK signaling pathway [31]. Indeed, in silico analyses have shown that these nutrient-sensing pathways are enriched in those processes regulated by miR-17 and miR-20a [24]. This cluster has also been linked to lipid metabolism, being differentially expressed in coronary artery disease (CAD) subjects, as miR-17 and miR-20a were upregulated and other members of the cluster were downregulated [22]. It has also been positively associated with hyperlipidemia and total cholesterol levels [22]. A recent study found that circulating levels of miR-92a were increased in patients with hypertension and were correlated with atherosclerosis markers [32]. Another study found that miR-92a was upregulated in plasma exosomes from subjects with CAD and was suggested to be a potential biomarker for atherosclerosis diagnosis [33]. miR-17 has been found to significantly increase atherosclerosis in patients [34]. miR-17 is also increased in human acute myocardial infarction subjects [35] and mice [36]. Thus, it could be suggested that TMAO may increase cardiovascular risk by increasing the expression of these microRNAs, which could be used as potential biomarkers of cardiovascular disease.

Interestingly, we found that TMAO significantly upregulated miR-20a-5p in the two cancer cell lines used in our experiments (THP-1 and HepG-2), while it did not change in primary macrophages or murine liver organoids, suggesting that TMAO could have a different impact depending on the health status and the presence of cancerous processes. The role of miR-20a in cardiovascular diseases is controversial; miR-20a plasma levels are significantly increased in patients who have suffered postinfarction heart failure, and have also been associated with left ventricular dilatation, suggesting that miR-20a could be a potential biomarker for left ventricular modeling in postinfarction patients [37]. Furthermore, ApoE−/− mice with miR-20a knockout exhibited less atherosclerotic formation. In addition, miR-20a promoted atherosclerotic development and reduced reverse cholesterol transport in these mice [38], suggesting that miR-20a inhibition could be a potential target in atherosclerosis therapy. On the other hand, an in vitro study showed that miR-20a was repressed when human aortic endothelial cells were treated with oxidized LDL particles, and overexpressed miR-20a protected cells from ROS generation, suggesting that miR-20a could protect aortic cells from inflammatory conditions and atherosclerotic development [39]. Furthermore, miR-20a is protective against myocardial ischemia/reperfusion injury, as miR-20a mimics promoted cardiomyocyte viability and decreased apoptosis [40]. Thus, as miR-20a behaves differently in cancer cell lines and primary cells, it could be suggested that TMAO may impair lipid homeostasis through miR-20a only in tumor cells. Further research would be needed to clarify the role of miR-20a in cardiovascular disease and atherosclerosis.

We only studied SERPINE1 in HEPG-2 cells, as it is not highly expressed in macrophages. SERPINE1 (also known as PAI-1: plasminogen activator inhibitor 1) is involved in normal blood clotting, as it acts as an inhibitor of tissue-type plasminogen activator and urokinase-type plasminogen activator. SERPINE1 is highly expressed in subjects with atherosclerotic plaques [26,27,41], and it is thought to promote atherogenesis due to its pro-thrombotic capacity and its ability to deposit fibrin in atherosclerotic lesions [26]. It has also been reported that SERPINE1 expression correlates with the thickness and severity of atherosclerosis in human arteries [26], and it is localized in endothelial cells, smooth muscle cells, and macrophages [27]. Indeed, SERPINE1 is localized in endothelial and smooth muscle cells in healthy atherosclerotic human arteries. In advanced atherosclerotic lesions, its expression not only increases but is also localized in macrophages [41]. Overexpression of SERPINE1 leads to a reduced remodeling capacity of smooth muscle cells because it inhibits plasmin production, activating metalloproteases needed for remodeling. Furthermore, different cytokines stimulate SERPINE1 production [42]. It has been reported to be linked to hepatic steatosis [43]. SERPINE1 plasma levels are elevated in non-alcoholic fatty liver disease (NAFLD) and metabolic syndrome, positively correlating with VLDL plasma levels, body mass index, and T2D [44]. Furthermore, different SERPINE1 polymorphisms have been associated with a higher risk of CVD [45]. In subjects with thrombotic disorders, SERPINE1 was regulated by plasma miR-30c, which directly inhibited it [46]. Our results suggest that TMAO exposure in the liver could increase cardiovascular risk by increasing SERPINE1 expression, both through its target microRNAs and independently of them, and SERPINE1 could constitute a potential predictor of metabolic-related diseases.

IL-12A belongs to the IL-6/IL-12 cytokine family, forming the IL-35 heterodimeric cytokine that plays a role in inflammatory diseases [47]. The IL-12 cytokine family is pro-inflammatory and essential for the progression of hypertension. These cytokines promote interferon-gamma secretion and Th immune response, which are essential for macrophage differentiation [28]. IL-12A polymorphisms have been associated with the risk of coronary artery disease, and it has been suggested that different components of IL-35 (such as IL-12A) could influence the progression of atherosclerosis [47]. IL-12A has also been found to be upregulated in myocardial infarction [48]. TMAO increased IL-12A expression in THP-1 cells at the mRNA and protein levels, especially in the first hours of exposure, suggesting that TMAO promotes inflammation and could promote macrophage differentiation and atherosclerotic plaque progression through IL-12A.

Since we found an upregulation of the microRNAs and their genes and proteins in response to TMAO, we may suggest that the effects of TMAO on SERPINE1 and IL-12A are not directly mediated by these microRNAs. SERPINE1 has been suggested to be directly targeted by some members of the miR-17-92 cluster—specifically, by miR-19a/b. However, it has also been suggested that miR-17 and miR-20a could indirectly inhibit SERPINE1 through PDLIM5 [29,30]. Similarly, it has been reported that miR17-92 suppresses IL-12 production in macrophages in genetically modified mice. The modulation of IL-12 by this cluster is mediated by PTEN and, consequently, by the PI3K-Akt-GSK3 pathway [49]. However, higher levels of plasma TMAO were correlated with higher levels of plasma pro-inflammatory markers in peritonitis, including SERPINE1 [50]. Similarly, plasma TMAO levels were positively correlated with plasma IL-12 levels in patients with common variable immunodeficiency [51]. Therefore, TMAO could increase these inflammatory markers independently of the miR-17-92 cluster.

The main limitation of our study is its descriptive nature. Further studies are needed to reveal the molecular mechanisms by which TMAO modulates the expression of the miR-17-92 cluster. Furthermore, we did not determine whether the TMAO-mediated regulation of SERPINE1 and IL-12 is directly or indirectly mediated by the miR-17-92 cluster. Although these constraints prevent us from concluding that TMAO modulates SERPINE1 and IL-12 through the miR-17-92 cluster, our results strongly suggest that the miR-17-92 cluster could play a role in the effects of TMAO on inflammation and cardiovascular health.

## 4. Materials and Methods

### 4.1. Cellular Models

HepG-2 and THP-1 cells were obtained from the American Type Tissue Collection (Barcelona, Spain). The cells were maintained in RPMI for THP-1 and in DMEM for HepG-2 (Lonza), with 10% fetal bovine serum (FBS) supplemented with glutamine and antibiotics (Cultek). THP-1 cells were differentiated into macrophages by incubating them with 50 ng/mL of phorbol 12-myristate-13-acetate (PMA) for 72 h (Sigma).

Peripheral blood mononuclear cells (PBMCs) were obtained from a young, healthy male donor. The study protocol was approved by the IMDEA Food Ethics Committee (PI-040, 5 March 2020, Madrid, Spain), and the donor provided signed informed consent. PBMCs were isolated with Lymphoprep™ (StemCell Technologies) following the manufacturer’s instructions and differentiated into macrophages as previously described in [15].

Murine livers were obtained and their organoids were differentiated as previously described in [15]. All procedures were carried out following the European Communities Directive 86/609/EEC guidelines. The protocols were approved by the Animal Ethics Committee (Proex 281/15 and Proex 282/15) of Ramón y Cajal Hospital (Madrid, Spain).

### 4.2. Treatments

HEPG-2 cells were treated with 6 µM of TMAO for 4, 8, and 24 h. THP-1 cells were treated with 6 µM of TMAO for 12 and 24 h. Murine liver organoids were treated with 6 µM of TMAO for 4, 8, and 24 h, while human primary macrophages were treated with 6 µM of TMAO for 12 h. Treatment doses and times were selected from dose– and time–response curves previously produced in [15].

### 4.3. microRNA-Enriched RNA Isolation and Amplification

Cells were lysed with Tripure (ROCHE, Madrid, Spain). RNA was isolated following the phenol:chloroform methodology adapted to enhance microRNA precipitation [15]. RNA concentration was measured using a NanoDrop 2000 (Thermo Scientific, Rockford, IL, USA), and RNA integrity was checked in 2% agarose gels.

Micro-RNA-enriched RNA was isolated from human primary macrophages using the miRCURY RNA Isolation Kit—Cell and Plant (Exiqon, Bionova, Madrid, Spain) following the manufacturer’s instructions.

miRNA expression levels were measured by real-time quantitative PCR (RT-qPCR). First, they were reverse-transcribed with the miScript Reverse Transcription II Kit (Qiagen, Madrid, Spain) and amplified with the miScript SYBR Green PCR Kit (Qiagen, Madrid, Spain), using a specific forward primer (Isogen Lifescience, Barcelona, Spain) and a universal reverse primer included in the kits. microRNA levels were calculated with the 2^−ΔΔCt^ method, using RNU6 and RNU43 for normalization.

### 4.4. Gene Expression Measurement

Gene expression levels were measured by real-time quantitative PCR (RT-qPCR). RNA was reverse-transcribed with the Prime Script Reverse Transcription Kit (Takara, CA, USA), and cDNA was amplified using the FastStart Universal SYBR Green Master (Roche, Barcelona, Spain). Specific forward and reverse primers were used (Isogen Lifesciences, Barcelona, Spain). Gene expression levels were calculated using the 2^−ΔΔCt^ method and normalized with RN18S and RPLP0.

### 4.5. Measurement of Protein Levels

Cells were lysed in ice-cold NP-40 lysis buffer, and total protein was quantified using the Pierce TM BCA Protein Assay Kit (Thermo Scientific, Rockford, IL, USA). Then, 50 µg of protein was separated by SDS–PAGE, transferred onto nitrocellulose membranes, and probed with the anti-IL-12A 1:500 anti-Serpine1 (Biorbyt) 1:2000, anti-β-actin (Abcam, Madrid, Spain) 1:5000 primary antibodies, and the appropriate HRP-conjugated secondary antibodies. Protein bands were visualized and measured via densitometric analyses (ImageJ). Protein quantification was normalized with β-actin.

### 4.6. Statistical Analyses

All experiments were performed in triplicate, and biological triplicates were included in each experiment. Additionally, technical duplicates were included in all RT-qPCR reactions. Inconsistent replicates were repeated. Gene and microRNA expression values were Log2-transformed to adjust the data to a normal distribution. The experiments included a control sample in each timepoint. Therefore, Student’s *t*-test was used to compare microRNAs, along with gene and protein expression values, between TMAO- and control-incubated samples at each timepoint. Statistical significance was set at *p* < 0.05. All analyses were performed using SPSS 19.0 (SPSS Inc., IBM Spain, Madrid, Spain).

## 5. Conclusions

In summary, we found that miR-17 and miR-92a—two members of the miR-17-92 cluster—were upregulated by TMAO, suggesting that TMAO modulates this cluster. The fact that miR-20a levels were not changed by TMAO means that TMAO could specifically modulate some members of the cluster, but not all, and that miR-20a could respond differently when there are cancerous processes. TMAO also increased the expression of two targets of inflammation and atherosclerosis—SERPINE1 and IL-12A—at the gene and protein levels. However, these inflammatory markers could be modulated by TMAO independently of the miR-17-92 cluster. Thus, it could be suggested that TMAO-mediated induction of the miR-17-92 cluster and of SERPINE1 and IL-12A could promote the development of inflammation and atherosclerosis.

## Data Availability

The data presented in this study are available upon request from the corresponding author.
